# A Ring Artifact Correction Method: Validation by Micro-CT Imaging with Flat-Panel Detectors and a 2D Photon-Counting Detector

**DOI:** 10.3390/s17020269

**Published:** 2017-01-30

**Authors:** Mohamed Elsayed Eldib, Mohamed Hegazy, Yang Ji Mun, Myung Hye Cho, Min Hyoung Cho, Soo Yeol Lee

**Affiliations:** Department of Biomedical Engineering, Kyung Hee University, Yongin, Gyeonggi 17104, Korea; m.deeb1@yahoo.com (M.E.E.); mohammed_hegazy_2012@yahoo.com (M.H.); ansdidwl@naver.com (Y.J.M.); myunghye.cho@raymedical.co.kr (My.H.C.); mhcho@khu.ac.kr (Mi.H.C.)

**Keywords:** ring artifact correction, flat-panel detector, photon-counting X-ray detector, correction vector, Gaussian filter

## Abstract

We introduce an efficient ring artifact correction method for a cone-beam computed tomography (CT). In the first step, we correct the defective pixels whose values are close to zero or saturated in the projection domain. In the second step, we compute the mean value at each detector element along the view angle in the sinogram to obtain the one-dimensional (1D) mean vector, and we then compute the 1D correction vector by taking inverse of the mean vector. We multiply the correction vector with the sinogram row by row over all view angles. In the third step, we apply a Gaussian filter on the difference image between the original CT image and the corrected CT image obtained in the previous step. The filtered difference image is added to the corrected CT image to compensate the possible contrast anomaly that may appear due to the contrast change in the sinogram after removing stripe artifacts. We applied the proposed method to the projection data acquired by two flat-panel detectors (FPDs) and a silicon-based photon-counting X-ray detector (PCXD). Micro-CT imaging experiments of phantoms and a small animal have shown that the proposed method can greatly reduce ring artifacts regardless of detector types. Despite the great reduction of ring artifacts, the proposed method does not compromise the original spatial resolution and contrast.

## 1. Introduction

The appearance of ring artifacts is a great concern in a three-dimensional (3D) computed tomography (CT) based on a two-dimensional (2D) X-ray detector, also called a cone-beam CT when a divergent X-ray source is used. In a cone-beam CT, a 2D X-ray detector is used to acquire the projection data while the gantry holding the X-ray source and detector pair moves on a circular scan path or the imaging object rotates on the central axis connecting the X-ray source and the detector. Since the pixels of many 2D flat-panel detectors (FPDs) are smaller than that of discrete detectors in typical medical CTs, the signal-to-noise ratio (SNR) of a cone-beam CT is lower than a medical CT, which inevitably makes the scan time of a cone-beam CT longer than a medical CT. The long scan time of a cone-beam CT makes it difficult to correct the non-uniformity of detector sensitivity over a 2D detector since the non-uniformity of detector sensitivity may fluctuate during the scan [1,2]. In addition to the long scan time, the low X-ray exposure of a cone-beam CT further complicates a flat-field correction to reduce ring artifacts [3]. A simple flat-field correction for a 2D X-ray detector along with a dark-current correction may not work well for multiple projection images taken at the cone-beam scan. Even with a flat-field acquisition with a long exposure time or a frame averaging, ring artifacts often persist in cone-beam CT images.

For a cone-beam CT, either an integrative FPD or a photon-counting X-ray detector (PCXD) can be used to acquire 2D projection data [4]. FPDs have readout circuits to produce electric signals whose amplitudes are proportional to the incoming X-ray intensity. The detector elements in the readout circuit may have a slightly different sensitivity element by element, which can produce ring artifacts in the CT images [1]. FPD-based cone-beam CTs have been more popular than PCXD-based cone-beam CTs. However, PCXD-based cone-beam CTs are expected to find many applications, especially for spectral X-ray imaging [5,6]. Spectral images taken with a Si PCXD [7] and a CdTe PCXD [2] have recently been reported. Recent developments of photon-counting readout chips for CdTe sensors, which can detect X-ray photons with energy higher than 100 keV, aim to perform 3D spectral imaging for human studies in the near future [8,9,10]. In X-ray imaging with a PCXD, low-intensity X-ray beams are used to avoid output pulse pileups [11,12]. In low dose imaging with a long scan time, the response of a PCXD may fluctuate due to the temperature fluctuations in the detector making the photon counts erroneous [2]. There are other factors that make the photon counts fluctuating, like electric noise [13] and Poisson noise in counting the incoming X-ray photons.

There have been many reports on ring artifact correction methods. Ring artifact correction can be made either on the sinogram domain [14,15,16,17,18,19] or on the CT image domain [20,21,22]. In the sinogram, the non-uniformity appears as stripes; hence, most sinogram domain approaches estimate the non-uniformity exploiting the feature of stripes in the sinogram. In the image domain approaches, the concentric ring components in the CT images are estimated and are subtracted from the original CT image. There were a few reports on the ring artifact corrections on the 2D projection images in a cone-beam CT [23,24].

In this paper, we introduce a novel ring artifact correction method for a cone-beam CT. For experimental verification of the proposed method, we used two types of 2D detectors, one integrative FPDs and the other a PCXD. Because of different mechanisms to detect X-ray photons and different noise characteristics between a FPD and a PCXD, the ring artifacts may show some different behaviors from each other. We have developed a universal ring artifact correction algorithm that works well in both types of X-ray detectors.

## 2. Materials and Methods

**Figure 1:**
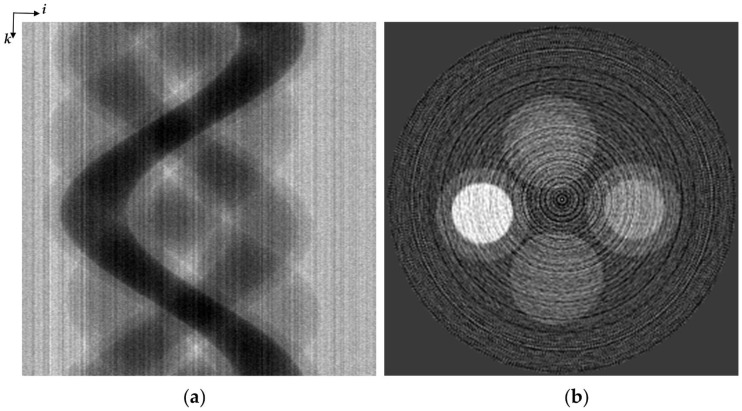
An example of stripe patterns in the sinogram and ring artifacts in the CT images obtained with the flat-field correction. (**a**) A sinogram generated from flat-field corrected projections of a four-tube contrast phantom acquired by a Si PCXD with an energy threshold of 14 keV. (**b**) The CT image reconstructed from the projection data shown in Figure 1a.

Non-uniformity of the sensitivity over the detector elements in a FPD or a PCXD makes stripe patterns in the sinogram of a slice. Figure 1a is an example of strong stripe patterns in a sinogram generated from flat-field corrected projections taken by a PCXD. If the non-uniformity is totally corrected in the flat-field acquisition procedure, then the stripe patterns can be completely removed by the flat-field correction. However, the flat-field image, also called the white image taken without placing any object between the X-ray source and the detector, has uncertainty at every pixel due to the detector noise and photon-counting statistics. Therefore, sinograms often have noticeable stripes even after the flat-field correction as demonstrated in Figure 1a. The stripe patterns make ring artifacts in the CT image as shown in Figure 1b.

### 2.1. Defective Pixel Correction

Defective pixels of a 2D X-ray detector show extremely low or high response. Since defective pixels show non-linear behavior with respect to the input X-ray intensity, the flat-field correction would not work for the defective pixels. Defective pixels may appear as a single pixel, a cluster of pixels, or vertical and horizontal lines. Since the position of defective pixels is fixed in all the projection data, we can identify the defective pixels by searching the pixels that show extremely low or high response in all the projection data. From the 3D stack of flat-field corrected projection data P(i,j,k), we compute the mean projection image by taking the mean value at each pixel over all the projection data along the view angle *θ*:

(1) $$\bar{P}(i,j)=\frac{1}{N_{\theta }}\sum_{k=1}^{N_{\theta }}P(i,j,k)\;\;i=1,2,....N_{X},\;\;j=1,2,....N_{Y}$$

where *N_θ_* is the number of views, and *N_X_* × *N_Y_* is the number of pixels of the projection images. Defective pixels are then identified by choosing the pixels whose values are lower or higher than global thresholds chosen empirically in consideration of the average pixel value over the projection data. However, if there are any highly attenuating objects near the rotation axis, some normal pixels may be identified incorrectly as defective pixels. In that case, the defective pixels can be identified by searching extremely low responding pixels from the flat-field images and extremely high responding pixels from the dark-current images.

With the position of each defective pixel identified, the defective pixel values in the 3D projection data P(i,j,k) are substituted with the ones computed from the neighboring normal pixels through 3D interpolation. The window size of the 3D interpolation is chosen so as to include more normal pixels than defective pixels, especially for clustered defective pixels. The algorithm starts with a small 3D window centered at the defective pixel of interest, and the 3D window size is progressively enlarged in all directions until the number of normal pixels inside the window becomes greater than that of defective pixels. Any other neighboring defective pixels inside the 3D window are rejected in the 3D interpolation.

### 2.2. Mis-Calibrated Pixel Correction

Mis-calibrated pixels of a 2D X-ray detector show a slightly larger or a smaller response than normal pixels making stripe patterns in the sinogram. If not corrected properly, mis-calibrated pixels generate ring artifacts in the CT images. After defective pixels have been corrected in the 3D projection data at all view angles, mis-calibrated pixels are corrected in the sinogram domain, i.e., row by row of the 2D X-ray detector. At the *j*-th row of the detector, a sinogram is made by taking the same rows from every projection image at different view angles after making the defective pixel correction:

(2) $$S_{j}(i,k)=P_{C}(i,j,k)\;k=1,2,....N_{\theta }$$

in which *P_c_* is the projection images after the defective pixel correction. Then, the mean value at the *i*-th pixel is computed over all the view angles to obtain a 1D mean vector ***m****_j_*:

(3) $$m_{j}(i)=\frac{1}{N_{\theta }}\sum_{k=1}^{N_{\theta }}S_{j}(i,k).$$

**Figure 2:**
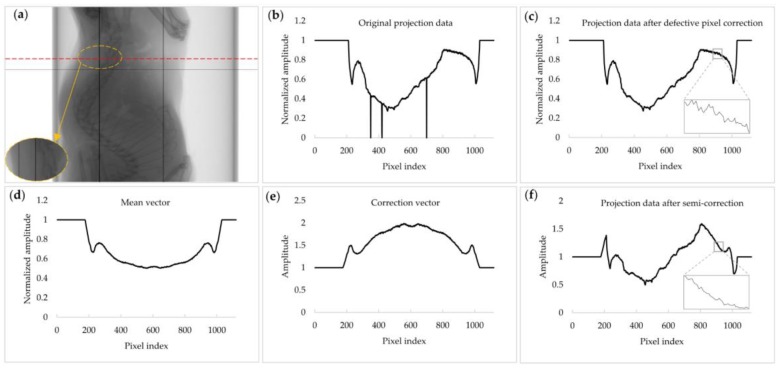
An example of mis-calibrated pixel correction. (**a**) A projection image of a rat taken with a FPD. The magnified region shows some defective pixels appearing as vertical lines. (**b**) The original projection data along the dashed line in Figure 2a. (**c**) The projection data after the defective pixel correction. (**d**) The mean vector. (**e**) The correction vector. (**f**) The projection data after the semi-correction.

The profile of the mean vector is expected to have a much smoother shape since it has an average shape of all projection data on the sinogram. An example of the projection data of a rat taken with the Dexela FPD is shown in Figure 2a. It has some defective pixels. After the defective pixel correction, the projection data is changed as shown in Figure 2c. Figure 2d shows the mean vector over the whole sinogram. The correction vector ***C**_j_* is then computed as follows:

(4) $$C_{j}(i)=\frac{1}{m(i)}.$$

The profile of the correction vector is shown in Figure 2e. The sinogram is then corrected by

(5) $${\tilde{S}}_{j}(i,k)=S_{j}(i,k)C_{j}(i).$$

**Figure 3:**
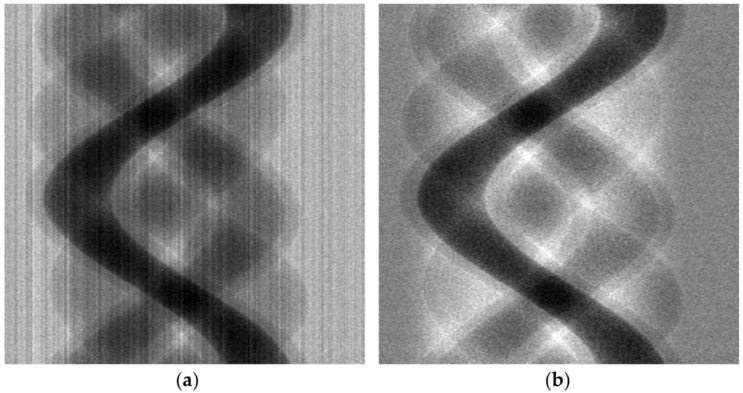
An example of mis-calibrated pixel correction in the sinogram acquired with a PCXD. (**a**) Before the correction. (**b**) After the semi-correction.

Figure 2f shows the corrected projection data. In the magnified windows in Figure 2c,f, it can be seen that the small ripples in Figure 2c are greatly reduced in Figure 2f, which would result in the reduction of stripe patterns in the sinogram. However, the original shape of the projection data is somewhat distorted in Figure 2f. Distortion of the projection data will introduce a contrast change in the reconstructed CT image. Figure 3 demonstrates the reduction of stripe patterns in the sinogram taken with a PCXD. Since the image reconstructed from ${\tilde{S}}_{j}(i,k)$ may have a contrast change, it will henceforth be called a semi-corrected image. Figure 2 shows the correction procedures step by step for a particular row (along the dashed line in Figure 2a) in the sinogram. The magnified region in Figure 2a shows some defective pixels appearing as vertical lines. In Figure 2, a projection data acquired with a FPD was used rather than a PCXD since the projection data acquired with a PCXD was too noisy to demonstrate the correction effects clearly. However, Figure 3 shows the sinograms acquired with a PCXD before and after the correction.

### 2.3. Contrast Compensation

**Figure 4:**
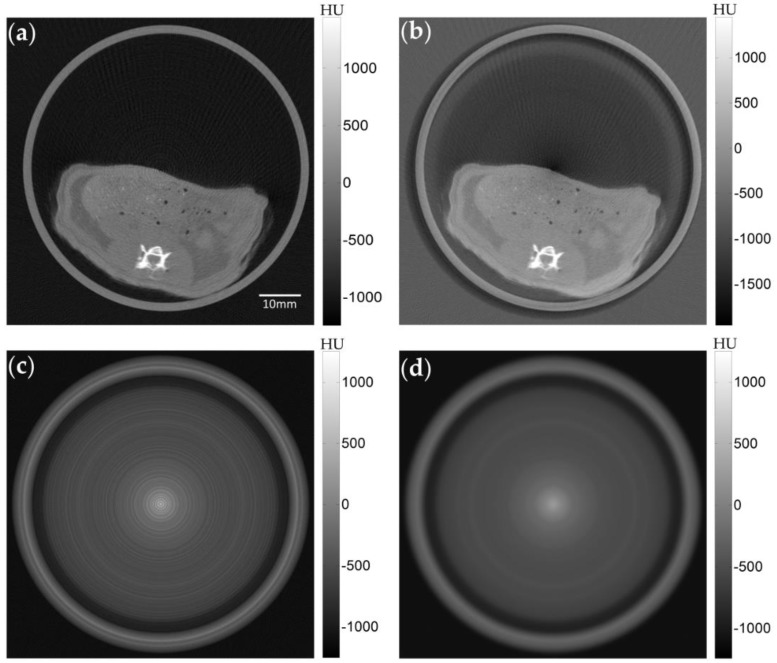
An example of semi-correction on a rat image taken with a FPD. (**a**) The original image obtained with flat-field correction. (**b**) The semi-corrected image obtained with applying the correction vector to the sinogram. (**c**) The difference image between (**a**) and (**b**). (**d**) The difference image after applying the Gaussian filtering.

The summation of the sinogram elements along the projection angles in computing the correction vector may cause a contrast change in the CT image since the correction vector are affected by the imaging object itself. To compensate the contrast change in the semi-corrected image caused by the mis-calibrated pixel correction, the difference image D(l,m) is computed between the original CT image $I_{0}(l,m)$, reconstructed from the original sinogram, and the semi-corrected CT image $\tilde{I}(l,m)$ reconstructed from ${\tilde{S}}_{j}(i,k)$:

(6) $$D(l,m)=I_{o}(l,m)-\tilde{I}(l,m)\;\;l=1,2,.....L,\;m=1,2,.....M$$

in which *L* × *M* is the CT image size. The difference image will contain thin ring patterns due to the mis-calibrated pixels and the component of the contrast change. Figure 4a shows an example of the original ring-artifact-corrupted image, and Figure 4b shows the image reconstructed after the semi-correction. The contrast change, particularly at the central region, can be seen in Figure 4b. Figure 4c shows the difference image between Figure 4a,b. To recover the contrast component, a Gaussian filter is chosen to smooth out the difference image. The smoothing level is based on the severity of the ring artifact intensity in the difference image. In most cases, the Gaussian filter size was 15 × 15 with a standard deviation of 10 pixels. The resultant filtered image $D_{G}(l,m)$, shown in Figure 4d, has rings that are much less intensive and represents the contrast change in the semi-corrected CT image. The summation of the Gaussian filtered difference image and the semi-corrected image makes the final image:

(7) $$I(l,m)=\tilde{I}(l,m)+D_{G}(l,m).$$

**Figure 5:**
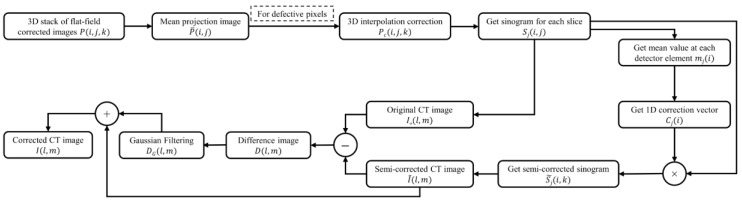
The procedures of the ring artifact correction.

The entire procedure of the ring artifact correction is summarized in Figure 5.

### 2.4. Micro-CT Imaging Experiments

To experimentally verify the proposed method, raw projection data have been acquired with a lab-built micro-CT system. The micro-CT consists of a micro-focus X-ray tube and an X-ray detector facing each other, and a rotation stage in between them to make the CT scan. All the components are placed on an optic table with a size of 110 cm × 80 cm. For X-ray detection, two FPDs and one PCXD were used alternately. The micro-focus X-ray tube (Series 5000, Oxford instruments, UK) is a sealed tube with a fixed molybdenum anode having a beam angle of 23° and a 127 µm thick beryllium exit window. The nominal focal spot size of the X-ray tube is 50 µm. Two FPDs from different vendors have been used. One is a CMOS FPD (Dexela 1512, Perkin Elmer, Santa Clara, CA, USA) with a 1944 × 1536 active matrix of transistors and photodiodes with a pixel pitch of 74.8 µm, and the other is a CMOS FPD (C7942, Hamamatsu, Japan) with a 2240 × 2240 active matrix of transistors and photodiodes with a pixel pitch of 50 µm. Both the FPDs use CsI:Tl as a scintillator. The scintillator thicknesses of the Dexela and Hamamstsu detectors are 600 µm and 200 µm, respectively. For a photon-counting detector, a PCXD (XRI-UNO, X-Ray Imatek, Barcelona, Spain), with 256 × 256 elements and a pixel pitch of 55 µm, has been used. The PCXD has a Medipix2 CMOS readout circuit with a 14 bit dynamic range. The PCXD uses 300-µm-thick Si as a photoconductor, which has an energy range of 4–25 keV with a 55% drop-off at 15 keV. The active area of the PCXD is 14.1 mm × 14.1 mm.

In the CT imaging with the Hamamatsu FPD, the tube voltage and current were 80 kV and 0.3 mA, respectively, and the detector integration time was 1000 ms. The source-to-object distance (SOD) and object-to-detector distance (ODD) were 321 mm and 111 mm, respectively. For the Dexela FPD, the tube voltage and current were 50 kV and 0.5 mA with the detector integration time of 200 ms. The source-to-object distance (SOD) and object-to-detector distance (ODD) were 585 mm and 85 mm, respectively. In the CT imaging with the PCXD, the tube voltage and current were 50 kV and 0.4 mA, respectively. The detector count time was 1000 ms at each view, and the threshold energy of the detector was 14 keV. The SOD and ODD were 321 mm and 96 mm, respectively. In all cases, the projection data were flat-field corrected using a single frame of the white image. Scanning was done over 360° with a step angle of 1° for the Hamamatsu FPD and PCXD, and 0.8° for the Dexela FPD.

Using the Dexela FPD, we took CT images of a rat post mortem under guidance of the institutional review board. We also took CT images of a QUART test phantom (DVT_AP, Quart GmbH, Zorneding, Germany) using the Dexela FPD. To take CT images of a small contrast phantom with a diameter of 40 mm and a height of 80 mm, we used the C7942 FPD and the PCXD. The contrast phantom consists of four tubes with a diameter of 3 mm, which contain 0.5 M and 0.05 M iodine solutions and 0.5 M and 0.05 M copper sulfate solutions.

## 3. Results

**Figure 6:**
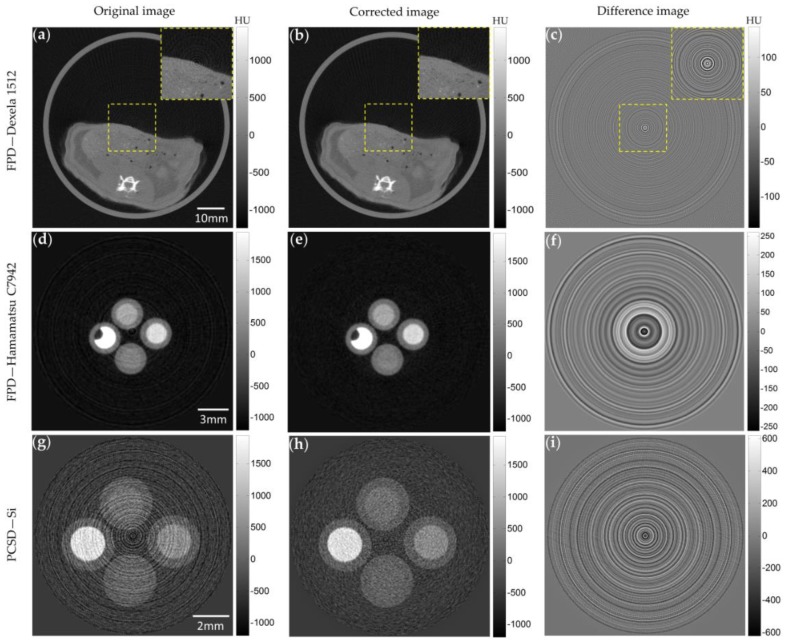
Ring artifact correction results. The first row shows the images of a rat taken by the Dexela 1512 FPD, and the second and third rows show the images of a contrast phantom taken by the C7942 FPD and the PCXD, respectively. The first column shows the original images obtained with applying the flat-field correction, and the second column shows the images corrected by the proposed method. The third column shows the difference images between the original and corrected images.

Figure 6 shows the images taken with the micro-CT with different detectors. The first, second, and third rows show the images of the rat abdomen taken with the Dexela 1512 FPD, the images of the contrast phantom taken with the C7942 FPD, and the images of the same contrast phantom taken with the PCXD, respectively. The first, second, and third columns show the original images obtained with flat-field correction, the images corrected by the proposed method, and the difference images between the original and the ring-artifact-corrected images, respectively. As can be seen from the figures, the image taken with the PCXD shows much stronger ring artifacts than those taken with the FPDs. In all the three cases, the proposed method has reduced the ring artifacts greatly, and the difference images show only the ring patterns, suggesting that the original contrasts are well preserved.

**Figure 7:**
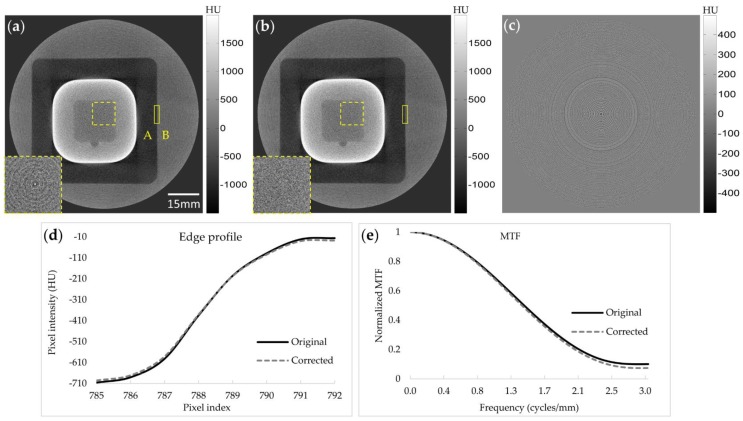
Ring artifact correction on a QUART phantom image taken with the Dexela 1512 FPD and evaluation of MTFs. (**a**) The original CT image obtained with the flat-field correction. (**b**) Its corrected image. (**c**) The difference image between (**a**) and (**b**). (**d**) Edge profiles at the rectangular ROI indicated by the solid lines in (**a**) and (**b**). (**e**) MTFs at the same ROI as in (**d**). Magnified images of the dashed ROI show effective ring artifact reduction in the uniform region.

Since we applied the Gaussian filter to the difference image, between the original uncorrected image and the semi-corrected image, the resultant final CT image may show some degradation in spatial resolution. To evaluate the spatial resolution after the ring artifact correction, we applied the proposed algorithm to the QUART phantom data taken by Dexela 1512 FPD. Figure 7a,b show the original and corrected images, respectively, and Figure 7c shows the difference between them. The rectangular region of interest (ROI) indicated by the solid lines is chosen to measure the edge profile and the modulation transfer function (MTF) of the CT images before and after the ring artifact correction. Visual inspection of the images before and after the correction, shown in Figure 7a,b, finds no difference in terms of spatial resolution. In the difference image shown in Figure 7c, no residuals of the edge structure of the phantom appear with the ring patterns appearing only. This suggests that the Gaussian filtering of the difference image does not have any significant negative effects on the spatial resolution. Magnifications of the ROI in a uniform region, indicated by the dashed line in Figure 7a,b, demonstrate a great ring artifact reduction. We measured SNRs at this ROI, and we summarize them here in Table 1. After the ring artifact correction, the SNR in the uniform region improved due to the ring artifact reduction. Figure 7d,e show the edge profiles and MTFs, respectively, computed at the ROI, with a size of 101 × 8, indicated by the solid lines in Figure 7a,b. The ROI is positioned around the interface between a low-attenuating substance (denoted as A in the figure) and a highly attenuating substance (denoted as B in the figure). The edge profiles were taken horizontally inside the ROI row by row, and the averaged edge profile was computed from them. For better visualization, the edge profiles have been fitted to smooth curves using the spline interpolation. We computed the MTF curves by first taking derivative of the average edge profile and then taking Fourier transform of the derivatives. We also computed contrast-to-noise ratios (CNRs) at Regions A and B, and we summarize them here in Table 1. The CNR has also been improved due to the reduction of ring artifacts.

## 4. Discussion

We applied the proposed ring artifact correction method to both types of 2D X-ray detectors, two scintillator-based integrative FPDs, and a semiconductor-based PCXD. We have observed that the ring artifact generation in the PCXD is dependent on the tube voltage and the energy threshold, which makes it more difficult to remove ring artifacts from the images taken with a PCXD. Longer exposure time of the PCXD also complicates the ring artifact reduction since PCXD outputs may fluctuate over the acquisition time in the CT scan.

The proposed method has shown significant reduction of ring artifacts, regardless of X-ray detector types. Gaussian filtering of the difference image may cause blurring of the original structure; however, it has little effect on the spatial resolution of the final image since the pixel intensities of the difference image are far lower than those of the semi-corrected image. In addition, the contrast change in the difference image is slowly varying all over the image space; hence, the Gaussian filtering recovers the original contrast well by blurring the ring artifacts only.

As compared to the previous methods, the proposed method has achieved acceptable results in the case of imaging with the FPDs. Correcting the stripe artifacts in the sinogram domain using wavelet-Fourier filtering, residual ring artifacts may persist in some cases and wave patterns may appear as reported in [14]. In another study based on the sinogram-domain approach [19], an improved ring artifact removal performance was reported as compared to wavelet-Fourier filtering, but it had residual ring artifacts too when it was applied to the uniform phantom imaging. In the other method [16], summation of the sinogram along the angular direction followed by smoothing was employed to estimate the non-uniformity of the detector response. Based on our observations, we think that making a correction vector with smoothing may lead to some contrast change, particularly around the edge region. In the proposed method, the contrast change has been compensated completely.

Computational burden of the proposed method is minimal, as compared to the 3D image reconstruction, since most computation is based on simple addition and multiplication. Despite the simplicity of the algorithm, the proposed method works well in both the phantom and animal imaging, regardless of detector type.

## 5. Conclusions

We show here the experimental results of ring artifact reduction in micro-CT imaging based on a FPD or a PCXD for X-ray detection. Regardless of detector type, integrative or photon-counting, the proposed method reduces the ring artifacts greatly without compromising the original spatial resolution and contrast. We expect the proposed method can also be used for a clinical cone-beam CT based on a 2D X-ray detector.

## Figures and Tables

**Table 1:** Signal-to-noise ratios (SNRs) at the dashed region of interest (ROI) and contrast-to-noise ratios (CNRs) between Regions A and B in Figure 7.

	SNR(dB)	CNR(dB)
Original image	−10.4	8.3
Corrected image	−9.3	9.1

## Abbreviations

The following abbreviations are used in this manuscript:

CT: Computed tomography
FPD: Flat-panel detector
PCXD: Photon-counting X-ray detector
Si: Silicon
CdTe: Cadmium telluride
ROI: Region of interest
MTF: Modulation transfer function
